# Radiological Findings Post Autologous Fat Grafting and Breasts Procedures: Avoiding False Positive Results

**DOI:** 10.1111/ans.70583

**Published:** 2026-03-13

**Authors:** Eduardo de Faria Castro Fleury

**Affiliations:** ^1^ Department of Radiology Faculdade de Ciências Médicas da Santa Casa de Misericórdia de São Paulo São Paulo Brazil

**Keywords:** breast cyst, fat necrosis, magnetic resonance imaging, mammography, ultrasonography

## Abstract

Oil cysts and fat necrosis are common complications of breast trauma. With the increased availability and sophistication of breast imaging, fat necrosis has become a frequent and typically benign finding in radiological practice. Nonetheless, oil cysts and fat necrosis can elicit an undesirable, persistent inflammatory response. Autologous fat grafting (AFG) is now widely used for breast augmentation, but one of its major complications is the development of oil cysts and calcifications. Oil cysts and fat necrosis demonstrate characteristic appearances on mammography, magnetic resonance imaging (MRI), and ultrasound, and their typical evolutionary patterns are pathognomonic for benignity. However, complications may result in imaging features that mimic suspicious lesions and warrant further diagnostic work‐up. This review illustrates the imaging complications of fat necrosis at each evolutionary phase and discusses their clinical implications and significance for diagnosis and patient management.

## Graphical abstract

This article highlights the importance of understanding the imaging features and complications of fat necrosis and oil cysts to improve diagnostic accuracy and clinical management. A rational diagnostic sequence—emphasizing comparison with prior imaging, careful evaluation of morphological changes, and judicious use of biopsy—can reduce unnecessary invasive procedures and avoid related patient morbidity.

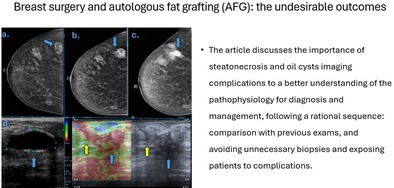

**Abbreviations**
AFG: Autologous fat grafting
BII: breast implant illness
BMI: body mass index
BoNTA: botulinum Toxin‐A
CDF: concentrated deoiled fat
FGF: fibroblast growth factor
M1‐type: inflammatory macrophages
M2‐type: anti‐inflammatory macrophages
MRI: magnetic resonance imaging
PDGF: platelet‐derived growth factor
TGF‐β: transforming growth factor‐β
TME: tumor microenvironment
VE: vitamin E
VEGF: vascular endothelial growth factor

## 1 Introduction and Background

Oil cysts and fat necrosis are frequent complications of breast trauma [1]. With advances in imaging for both diagnostic and screening purposes—and with the growing number of therapeutic and aesthetic breast procedures and percutaneous biopsies—fat necrosis has become a common finding in daily radiological practice. Fat necrosis is a benign condition resulting from injury to fatty breast tissue. As fat cells die, they release lipid content that forms a sac‐like collection of oily fluid known as an oil cyst. Despite its benign nature, oil cysts may provoke a persistent inflammatory response [1, 2, 3].

Autologous fat grafting (AFG) has emerged as an important option for breast augmentation in both aesthetic and reconstructive contexts, supported by technical refinements and scientific advances [4]. A primary complication of AFG is oil cyst formation, which occurs when non‐vascularized fat is injected to fill tissue defects [3].

In 1987, the American Society of Plastic Surgeons (ASPS) banned the use of autologous fat grafting (AFG) to the breasts because of concerns regarding safety and difficulties in radiographic interpretation [5]. This moratorium was reversed in 2009 after the ASPS Fat Graft Task Force evaluated the safety and efficacy of AFG. The ASPS 2019 procedural statistics report demonstrated a 37% increase in breast fat grafting compared with 2015 (24 892 vs. 18 135 procedures) [6]. However, comparison of the 2022 and 2021 statistics revealed a 4% reduction in AFG procedures (17 358 vs. 18 106) [7]. From 2023 to 2025, the ASPS did not publish annual statistics on breast fat grafting, which limits trend analysis and assessment of the potential impact of the COVID‐19 pandemic.

The main complications associated with AFG include oil cysts, fat necrosis, persistent pain, induration, calcifications, and the development of breast striae. Oil cysts and fat necrosis typically demonstrate characteristic and usually benign imaging features on mammography, magnetic resonance imaging (MRI), and ultrasound [3]. The typical appearance of an oil cyst is an oval, well‐circumscribed mass with heterogeneous content, including fat, fluid, and calcifications, without suspicious vascularity on MRI and ultrasound. Calcifications may range from coarse heterogeneous to dystrophic on mammography. However, complications related to AFG may occasionally mimic suspicious lesions, prompting further diagnostic evaluation [7]. The differential diagnosis of AFG‐related complications includes breast carcinoma, radial scars or complex sclerosing lesions, fibrocystic changes, and fibroadenomas in our practice.

### 1.1 Physiopathology and Imaging Findings of Oil Cysts and Fat Necrosis

Oil cysts result from the evolution of fat necrosis. Fat necrosis is a sterile inflammatory process divided into hyperacute, subacute, and late phases [8]. Each phase exhibits distinct pathophysiological mechanisms and imaging features [1, 2, 4].

### 1.2 Hyperacute Phase

**FIGURE 1:**
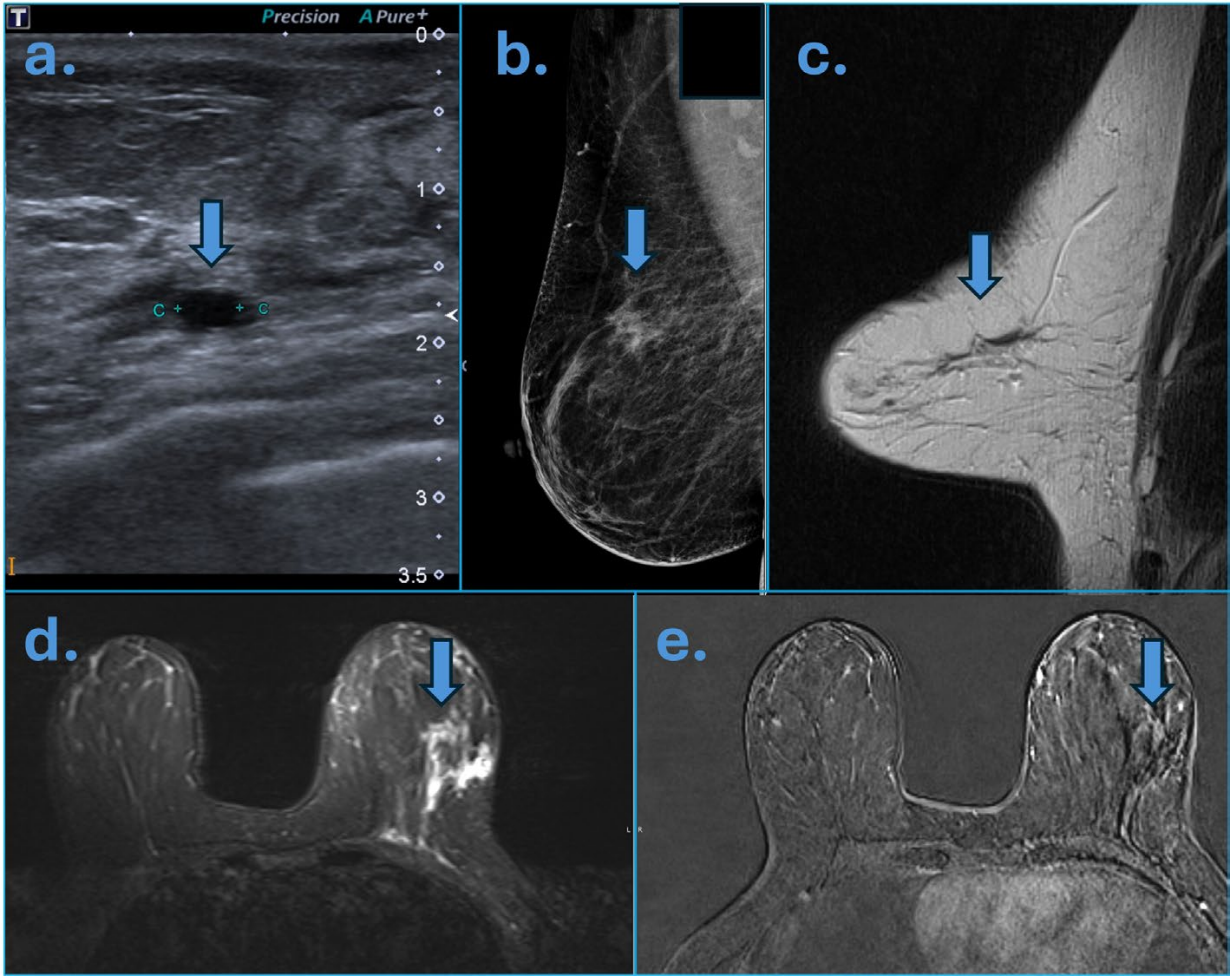
A 61‐year‐old woman after quadrantectomy of the left breast 2 weeks ago, with compromised margins. B‐Mode ultrasonography (a), oblique view mammography (b), sagittal PD sequence (c), axial T2 STIR sequence (d), and pos contrast T1 weighted sequence (e). A small fluid collection associated with architectural distortion pointed by the blue arrow (a). The mammography shows an architectural distortion with fatty content inside, black star (b). MRI shows the architectural distortion at the surgical site on PD (c), with high intensity signal on T2‐weighted sequence (d) and with no significant enhancement in post‐contrast sequences. The findings are compatible with scar tissue.

This phase begins immediately after adipocyte destruction and microvascular injury, leading to vasoconstriction and fluid transudation [8]. Early imaging—ultrasound, mammography, and breast MRI—often demonstrates architectural distortion at the surgical site. Color Doppler ultrasound and contrast‐enhanced MRI typically show increased perilesional vascularity [4, 8] (Figure 1).

### 1.3 Subacute Phase

Ongoing adipocyte destruction results in triglyceride breakdown and calcium‐mediated saponification of fatty acids. Residual lipids form vacuoles surrounded by lymphocytes and histiocytes, initiating a reparative process that may last weeks to months. A completely phagocytosed adipocyte is replaced by collagen, forming fibrous scar tissue. If the necrotic content persists, a fibrous capsule forms, creating an oil cyst.

**FIGURE 2:**
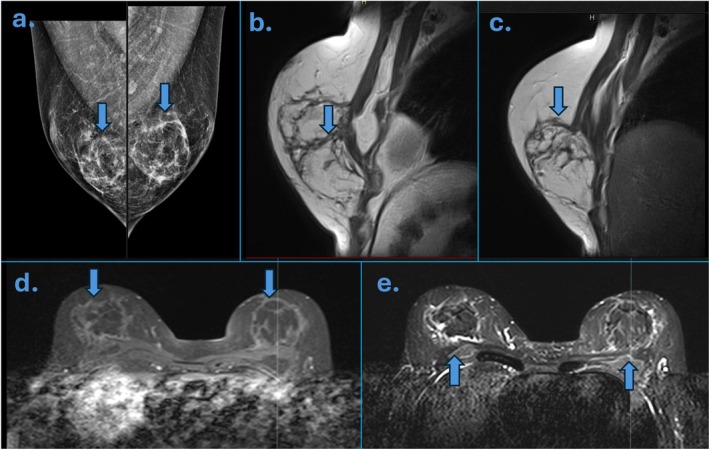
A 53‐year‐old woman, after bilateral mastopexy 4 had bilateral breast palpable lumps and pain. Oblique view mammography (a), sagittal PD sequence of the right (b) and left (c), axial SPIR sequence (d), and axial T2 STIR sequence (e). Mammography shows an architectural distortion (blue arrow) at the surgical site, with margins ill‐defined, and heterogeneous content (a). The sagittal PD sequences (b and c) show a pseudo‐capsular formation at the periphery of the architectural distortion, with medium intensity signal at T1 SPIR (d) and high intensity signal at T2 STIR (e). These findings show the ongoing bilateral fat necrosis in surgical sites, the early changes.

**FIGURE 3:**
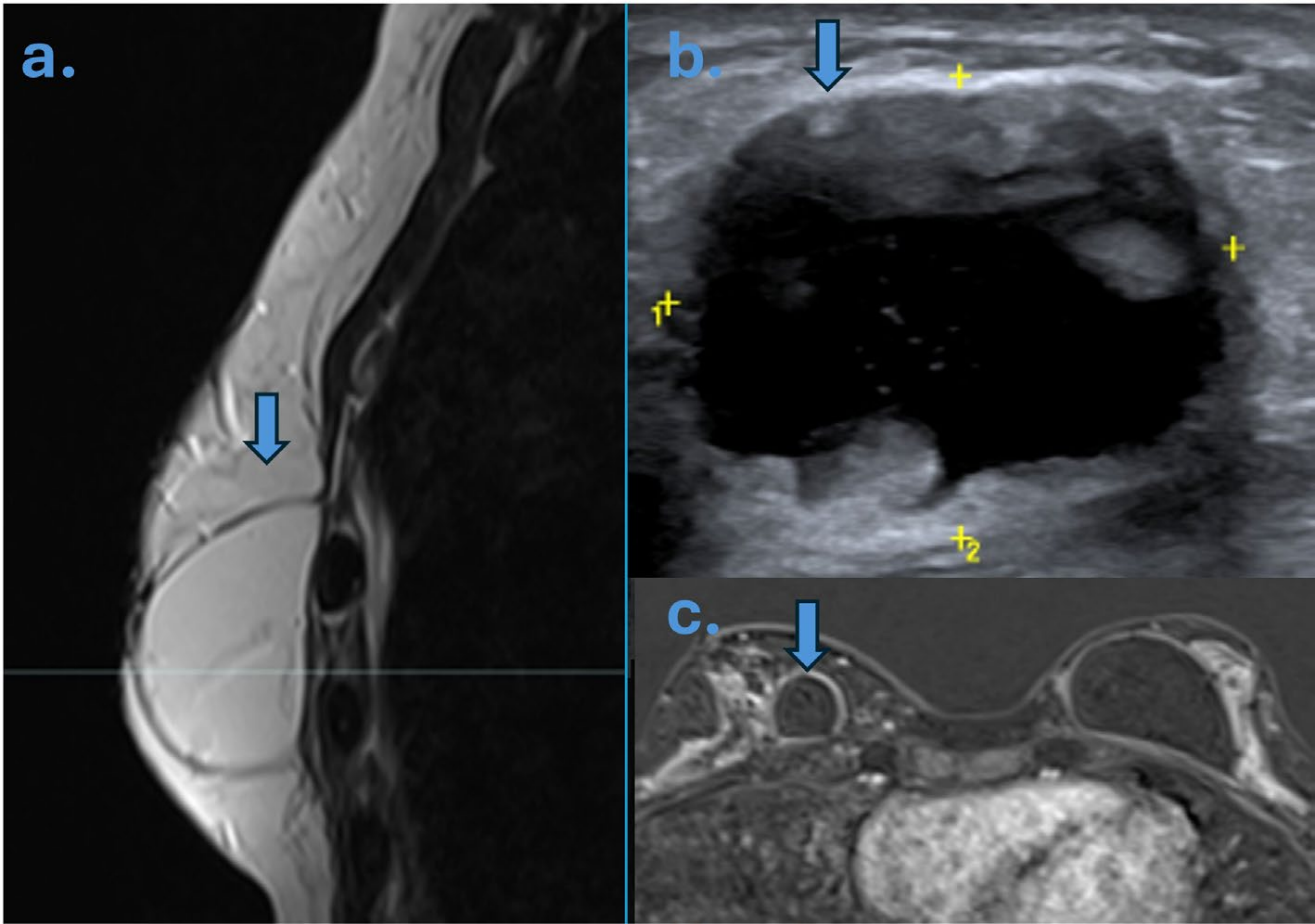
A 65‐year‐old woman, after explant surgery with bolus bilateral autologous fat grafting reconstruction for 5 weeks, presented with a palpable lump on the breast associated with pain. Sagittal PD sequence of the right breast (a), ultrasonography of the right breast (b), and pos contrast MRI of the bilateral breast (c). A heterogeneous fat‐containing collection with a thin capsule (blue arrow) is observed in the PD sequence (a). The ultrasonography shows a well‐defined heterogeneous mass, with suspended mobile echoes and heterogeneous tissue in the cyst wall (b). The post contrast sequence shows parietal enhancement of the cyst, indicating active Inflammation. The findings are compatible with an oil cyst in the early/medium stage.

Typical imaging findings—fat‐density round masses on mammography, heterogeneous cysts with mural nodularity on ultrasound, and non‐enhancing fatty cysts on MRI—are pathognomonic for benignity (Figures 2 and 3).

Inflammatory macrophages (M1‐type) initially phagocytose oil droplets, a process that may take weeks for a 1 mm droplet. When this response is insufficient, anti‐inflammatory macrophages (M2‐type) encapsulate the debris, forming a fibrous wall and halting absorption [4, 8].

### 1.4 Late Phase and Complications

**FIGURE 4:**
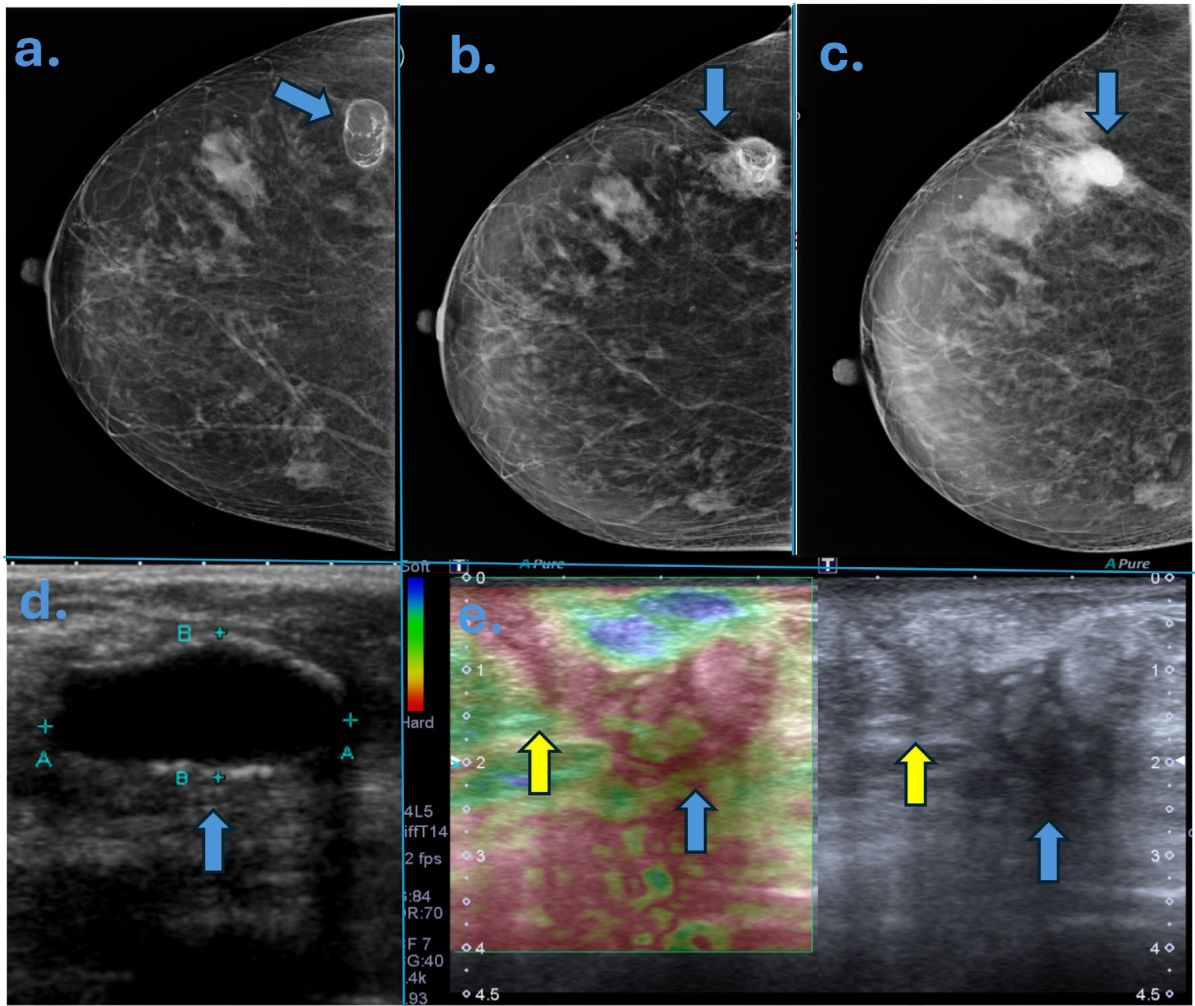
A 70‐year‐old woman after quadrantectomy of the right breast for cancer 15 years ago. Previous screening craniocaudal view mammography (a), current mammography (b), and post biopsy control (c), previous B‐Mode ultrasound (d), and post biopsy elastography ultrasound (e). The mammogram shows a typical calcified fat necrosis at the surgical site in screening series (a) and B‐Mode ultrasound (d), which evolves to an irregular dense mass at the medial aspect of the mass (b), considered as a suspicious lesion that was followed for breast biopsy. The post biopsy mammography shows an increase in the density in the periphery of the fat necrosis (c), hard at the elastography (e). Elastography also shows the needle tract extension of the process (yellow arrow). The pathology showed fat necrosis with acute Inflammation.

Late complications may occur 1–2 years after formation and present with variable clinical, physical, and imaging findings [4, 5, 6, 7, 8]. Depending on the host inflammatory response and degree of fibrosis, oil cysts may exhibit suspicious imaging features, including irregular or spiculated margins and increased vascularity on Doppler ultrasound or contrast‐enhanced MRI (Figure 4).

Mammography may show coarse wall calcifications—indicative of benignity—while ultrasound may demonstrate posterior shadowing. Chronic inflammation may lead to wall enhancement on dynamic MRI [4, 8].

Recent reports describe oil cyst rupture with surrounding inflammation, sometimes classified as suspicious according to BI‐RADS [8]. In our practice, biopsies of such lesions have occasionally exacerbated inflammation, extending along the needle tract and even mimicking acute breast infection (Figure 4).

Clinically, patients may present with a palpable mass, skin retraction, and inflammatory signs such as erythema, swelling, warmth, and pain.

## 2 Discussion

The primary concern in diagnosing complications of breast fat necrosis lies in the overlap of clinical and radiological findings with those of breast malignancy. The differential diagnosis includes breast cancer, phyllodes tumor, breast tuberculosis, and complex cystic lesions [1, 2, 3, 7].

Complications related to breast surgery are not new. In 1977, Baber and Libshitz published a seminal report describing an atypical case of bilateral fat necrosis following reduction mammoplasty. They emphasized the difficulty of distinguishing fat necrosis from malignancy based on imaging alone and predicted that the rising number of breast procedures would lead to increased cases and the need for clearer diagnostic criteria [1]. Nearly 40 years later, no standardized guidelines exist for the diagnosis or management of suspicious oil cysts.

With the rising number of breast surgeries and percutaneous biopsies—combined with increased life expectancy—the incidence of fat necrosis continues to rise. Although fat necrosis is benign, its complications can mimic malignant lesions on imaging [9, 10, 11].

**FIGURE 5:**
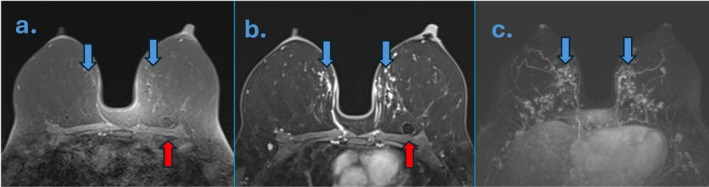
44‐year‐old woman after bilateral autologous fat grafting for aesthetical purposes for 6 months, presenting bilateral breast pain. Axial T1 SPIR sequence (a), axial T1 post contrast sequence (b), and MIP (multiplanar intensity projection) (c). The blue arrow shows uneven distribution of fat droplets with contrast enhancement, symmetric and bilateral, and the red arrow shows an oil cyst with parietal enhancement.

Autologous fat grafting (AFG) procedures have contributed to the increased frequency of oil cysts and fat necrosis. Yoshimura et al. demonstrated that fat particles larger than 2–3 mm cannot undergo complete graft integration, leading to necrosis. The authors attributed the development of oil cysts to the injection volume, technique, and characteristics of the recipient site. Small‐volume, multilayered microinjections reduce necrosis risk [4]. Complications often result from bolus injections or uneven distribution of fat droplets (Figures 3 and 5).

Autologous fat grafting (AFG) typically involves three steps: harvesting the lipoaspirate, processing it (most commonly by centrifugation), and reinjecting the purified fat into the target area [12, 13]. Centrifugation is performed in approximately 34% of AFG procedures and includes centrifuging the aspirate, wicking off the oil layer, draining the infranatant, and transferring the processed fat into a new syringe before injection into the target breast site. The primary aim of centrifugation is to concentrate the graft and remove tumescence fluid, blood, debris, and oil. It has also been hypothesized that eliminating blood and nonviable components may increase the concentration of adipose‐derived stem cells, thereby reducing graft resorption and attenuating the inflammatory response [14]. Alternative processing methods include the mesh technique, in which the lipoaspirate is placed over a porous mesh to allow gravity‐driven drainage of the aqueous component, and the Telfa technique, which involves rolling the aspirate over Telfa gauze to absorb the aqueous fraction. Both techniques have demonstrated results comparable to centrifugation [14]. Finally, active closed wash and filtration (ACWF) systems are defined by the immediate harvest of lipoaspirate into the device, followed by serial sterile washes with lactated Ringer's solution and direct separation from tumescent fluid and debris via an internal filter under vacuum suction. ACWF systems yield superior results compared with their passive counterparts, as well as with other traditional fat grafting techniques. The integration of all components of lipoaspirate processing into a single device provides several advantages, including reduced operative time, minimized tissue handling and exposure to outside air, and better control of pH and osmolarity, thereby promoting nourishment and neovascularization while avoiding an inflammatory response [15].

To enhance graft survival—given that 20%–80% of injected fat may be resorbed—some authors advocate the addition of adipose‐derived stem cells (“cell‐assisted lipotransfer”) [12, 16, 17]. Fat resorption occurs due to apoptosis and necrosis, predominantly in the central portion of the graft, where ischemia persists until neovascularization occurs [16]. Eto et al. described three concentric layers within the graft: the outer “surviving zone,” the central “necrotic zone,” and a middle “regenerative zone” [16].

Growth factors such as VEGF, PDGF, FGF, and TGF‐β, along with matrix metalloproteinases, facilitate angiogenesis and graft remodeling [13, 18, 19, 20]. New techniques—including vitamin E supplementation, compact fat grafting, botulinum toxin‐A (BoNTA) for muscle immobilization, concentrated deoiled fat (CDF), and biodegradable scaffolds—have shown potential for improving AFG survival [20, 21, 22, 23, 24, 25].

Despite these innovations, optimal patient outcomes still depend heavily on proper preoperative assessment, identification of risk factors (e.g., thrombotic history, immunosuppression, coagulopathies), and meticulous technique. Evidence suggests that small‐caliber cannulas, subcutaneous injection planes, and appropriate graft volumes reduce the risk of fat necrosis [26, 27].

Greer and Pestana, in their original study on the Lateral Breast Flap SLING technique, reported that grafted volumes exceeding 100–125 mL were associated with higher fat necrosis rates [28]. Obesity also increases risk; in their study, 50% of patients with BMI > 30 kg/m^2^ developed fat necrosis, compared with none in the non‐obese group. These findings align with a systematic review by Lazorle et al. which associated graft volumes > 100 mL with higher complication rates [29].

**FIGURE 6:**
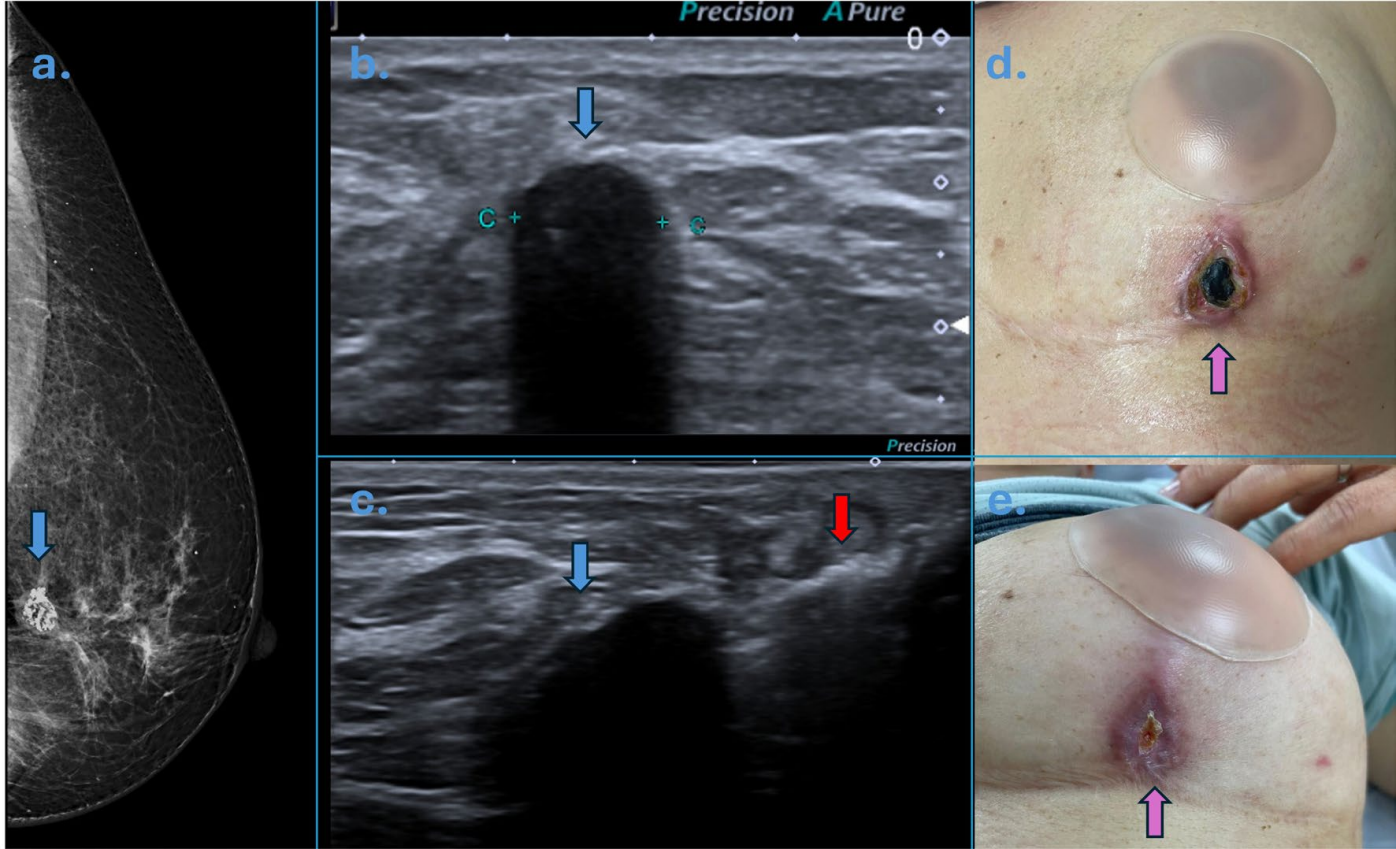
A 51‐year‐old woman after biopsy of a complicated late calcified fat necrosis. Cranio‐caudal mammography view (a), B‐Mode ultrasonography (b), B‐Mode biopsy guide ultrasonography (c), 4 weeks after the procedure (d), and 8 weeks after the procedure (e). The blue arrow shows a complicated calcified fat necrosis. After 1 week of the biopsy, the patient showed Inflammation of the biopsy tract (red arrow) extending to the skin (purple arrow), treated as an infection of the procedure site, but refractory to antibiotic therapy (a). After 4 weeks, we opted to treat with corticosteroids, where we observed remission of the lesion in 4 weeks.

The mechanisms behind chronic oil cyst dysregulation and the onset of acute inflammation remain unclear. Acute inflammatory complications may appear suspicious on imaging, often prompting biopsy (Figure 4). However, in our practice, biopsy may exacerbate the inflammatory response, particularly along the needle tract, with adverse cosmetic and clinical outcomes (Figure 6).

In such cases, previous imaging studies are invaluable. If earlier examinations demonstrate typical benign features, a conservative approach—including observation or a therapeutic trial of anti‐inflammatory medication or corticosteroids—is appropriate.

Oil cyst complications are particularly relevant following explant surgeries for breast implant removal. Many patients undergo explantation due to symptoms attributed to Breast Implant Illness (BII), a proposed immune‐mediated condition triggered by silicone gel bleed. To compensate for post‐explant volume loss—especially in retropectoral implants—surgeons frequently use large‐volume fat grafting. We have observed that some patients with late‐onset inflamed oil cysts experience a recurrence of symptoms similar to those initially attributed to BII.

Another area of concern is the potential association between AFG and atypical breast lesions. Ashkar et al. reported a case suggesting that adipose‐derived stem cells or growth factors might theoretically alter the breast microenvironment and promote premalignant cell proliferation [30]. However, causality remains unproven, and the reported case involved a 43‐year‐old woman—an age at natural risk for breast cancer—so AFG should not be considered an independent carcinogenic factor.

In 2024, Fleury et al. proposed that chronic inflammation associated with breast implant fibrous capsules may influence the tumor microenvironment, promoting metaplasia and possibly carcinoma [31]. The theory was supported by concepts such as silicone bleed, metaplastic adaptation to injury, T‐cell dysfunction, inhibitory cells within the tumor microenvironment, and parallels with the biology of medullary carcinoma [32, 33]. Chronic inflammation is a well‐established contributor to carcinogenesis in various diseases, including Barrett's esophagus–related adenocarcinoma, pancreatic ductal adenocarcinoma, and breast squamous cell carcinoma [34, 35, 36]. Fleury later described a possible association between silicone exposure and papillary breast lesions [36].

Diagnosing complicated fat necrosis and oil cysts is challenging due to their variable presentation and the lack of standardized guidelines—particularly in patients with prior breast cancer or additional risk factors. When clinical or imaging features are ambiguous, biopsy remains necessary to differentiate a suspicious oil cyst from malignancy [37, 38].

In our clinical practice, comparison with prior imaging is essential for risk stratification and management decisions, considering previous lesion features, primary tumor histology, and margin status [39].

Management of complicated oil cysts varies widely among institutions [37]. Surgical excision remains the traditional treatment for symptomatic fat necrosis [38]. Garsa et al. support conservative management with clinical and imaging follow‐up in the absence of concerning features, emphasizing patient reassurance—even for large lesions [40].

Alternative non‐surgical treatments include nonsteroidal anti‐inflammatory drugs (NSAIDs) administered for 1–2 weeks, sometimes in combination with analgesics. Pain may take up to 3 months to fully resolve [40]. Because oil cysts are associated with chronic inflammation, clinical treatment typically provides symptomatic relief only during the acute phase, and inflammatory symptoms may recur during follow‐up. Other reported techniques include ultrasound‐assisted liposuction and needle aeration [38, 41, 42].

Regarding the direct treatment of oil cysts, Yoshimura et al. proposed puncturing the cyst wall with a 14–18‐gauge needle to express its contents and stimulate phagocytosis [43]. However, in our experience, this intervention may exacerbate inflammation and should be avoided, particularly during the acute phase (Figure 6) [43, 44]. In our practice, before attempting aspiration of an inflamed oil cyst, nonsteroidal anti‐inflammatory drugs (NSAIDs) are administered to reduce inflammation and minimize the risk of a cascade of complications.

## 3 Conclusion

Breast reconstruction procedures involving fat grafting may be associated with a range of complications. Radiologists must be able to recognize and accurately document these findings to avoid false‐positive diagnoses and unnecessary invasive procedures. Familiarity with the imaging spectrum of fat necrosis and oil cysts supports appropriate classification, guides clinical management, and reduces the need for redundant biopsies. The involvement of a breast imaging specialist with experience in autologous fat grafting is essential to minimize unnecessary interventions and additional imaging examinations.

## Author Contributions

**Eduardo de Faria Castro Fleury:** conceptualization, investigation, funding acquisition, writing – original draft, methodology, validation, visualization, writing – review and editing, software, formal analysis, project administration, data curation, supervision, resources.

## Funding

The author has nothing to report.

## Ethics Statement

The author has nothing to report.

## Conflicts of Interest

The author declares no conflicts of interest.

## Data Availability

The data that support the findings of this study are available on request from the corresponding author. The data are not publicly available due to privacy or ethical restrictions.
